# Clinical effects of the Collaborative Care Model on pulmonary function and quality of life in postoperative patients with lung cancer

**DOI:** 10.12669/pjms.42.4.13278

**Published:** 2026-04

**Authors:** Pei Liu, Cuifang Liu, Weihua Liu, Xiaohui Qi, Chao Shi, Haozheng Cao

**Affiliations:** 1 Pei Liu, Department of Thoracic Surgery, Affiliated Hospital of Hebei University, Baoding 071000, Hebei, China; 2 Cuifang Liu, Department of Thoracic Surgery, Affiliated Hospital of Hebei University, Baoding 071000, Hebei, China; 3 Weihua Liu, Department of Thoracic Surgery, Affiliated Hospital of Hebei University, Baoding 071000, Hebei, China; 4 Xiaohui Qi, Department of Thoracic Surgery, Affiliated Hospital of Hebei University, Baoding 071000, Hebei, China; 5 Chao Shi, Department of Thoracic Surgery, Affiliated Hospital of Hebei University, Baoding 071000, Hebei, China; 6 Haozheng Cao, Department of Thoracic Surgery, Affiliated Hospital of Hebei University, Baoding 071000, Hebei, China

**Keywords:** Collaborative Care Model, Pulmonary function, Lung cancer, Postoperative, Quality of life

## Abstract

**Objective:**

To evaluate the clinical efficacy of the Collaborative Care Model (CoCM) in improving pulmonary function and quality of life in patients following lung cancer surgery.

**Methodology:**

This was a retrospective study. Eighty patients who underwent radical lung cancer resection at Affiliated Hospital of Hebei University’s Department of Thoracic Surgery between January 2022 to June 2025 were enrolled and randomly assigned to the observation group(n= 40, received CoCM in addition to the standard postoperative care based on the control group) and the control group(n= 40, received standard postoperative care) according to their choice of postoperative nursing approach. Pulmonary function parameters, arterial blood gas(ABG) parameters, activities of daily living(ADL) were compared between the two groups.

**Results:**

At seven days, one month, and three months post-intervention, pulmonary function parameters showed progressive improvement in both groups. The observation group demonstrated significantly greater improvements at each time point compared with the control group(*P<* 0.05, respectively). ABG parameters also improved over time in both groups, with the observation group exhibiting superior outcomes at all assessed intervals(*P<* 0.05, respectively). Both groups showed marked enhancement in ADL scores, with significantly greater improvements in the observation group(*P<* 0.05); Quality of life scores also increased significantly in both groups after one and three months, with the observation group achieving significantly more pronounced improvements than the control group(*P<* 0.05).

**Conclusion:**

The implementation of CoCM for patients recovering from lung cancer surgery can significantly enhance pulmonary function and ABG while also improving ADL and quality of life.

## INTRODUCTION

Lung cancer is one of the most common malignancies encountered in clinical practice. According to the GLOBOCAN 2020 report on global cancer incidence and mortality published by the International Agency for Research on Cancer, lung cancer ranks second worldwide in terms of incidence,^1^ accounting for 11.4% of all malignancies. Notably, both the incidence and mortality rates of lung cancer are the highest among all cancers in China.^2^ At present, lung cancer remains a major threat to human health and survival, underscoring the importance of comprehensive approaches to its diagnosis, treatment, and nursing care. With advances in medical technology, surgical resection is now the preferred treatment for patients with early- to mid-stage lung cancer.

Although radical lung cancer surgery can prolong survival, it often impairs postoperative pulmonary function. Studies have shown that it typically takes three to six months for patients to regain 60%-80% of their preoperative pulmonary function, significantly affecting their postoperative quality of life.^3^ In addition, surgery may trigger psychological stress responses and elevate psychological burden, further compromising patients’ recovery and overall well-being. Previous studies have demonstrated that pulmonary rehabilitation training can improve postoperative quality of life.^4^ However, conventional rehabilitation programs are predominantly educational and lack consistent supervision or active involvement by healthcare professionals. This absence of comprehensive, individualized support leads to suboptimal clinical outcomes.

Therefore, addressing the challenges associated with pulmonary rehabilitation, particularly ensuring that such training is accurate, efficient, and well-supervised, is an urgent clinical priority. The Collaborative Care Model (CoCM), is an innovative nursing framework that emphasizes a multidisciplinary approach.^5^ It involves active participation from healthcare professionals, patients, and family members, mobilizing available resources and enhancing patient engagement and adherence to care plans, with the ultimate goal of improving quality of life. In this study, CoCM was employed to guide postoperative pulmonary rehabilitation training in patients with lung cancer, aiming to evaluate its impact on postoperative pulmonary function and quality of life.

## METHODOLOGY

This was a retrospective study. Eighty patients who underwent radical lung cancer resection in the Department of Thoracic Surgery at Affiliated Hospital of Hebei University between January 2022 to June 2025 were enrolled in this study. Patients were randomly assigned to either the observation group or the control group using a random number method according to their choice of postoperative nursing approach, each group with 40 patients. The observation group received CoCM, while the control group received standard nursing care.

### Ethical approval:

The study was approved by the Institutional Ethics Committee of Affiliated Hospital of Hebei University (No.:HDFYLL-IIT-2023-036; Date: April 01, 2025), and written informed consent was obtained from all participants.

### Inclusion criteria:

- Aged 45-65 years old.
- Obtained the informed consent of participants’ families.
- Clinically diagnosed with lung cancer and underwent radical surgical resection at the age of 18 or above.
- Complete clinical records with adequate communication abilities.
- Availability of a designated caregiver with good communication skills.
- Provided informed consent and voluntarily agreed to participate in the study.
- No significant neuropsychiatric symptoms and able to cooperate with study procedures.

### Exclusion criteria:

- Presence of severe hepatic or renal dysfunction or other significant organic diseases.
- Diagnosis of other malignancies.
- Inability to cooperate with follow-up, or diagnosis of psychiatric disorders or cognitive impairment.

### Intervention Approaches:

All patients were treated immediately after admission. The control group received standard postoperative nursing care, which included health education, orientation to the hospital environment, information on the disease, surgical precautions, and guidance on healthy behaviors. Patients were instructed on pulmonary rehabilitation and encouraged to follow a staged rehabilitation plan. After discharge, patients were followed up for six months via a combination of telephone calls and outpatient visits to monitor their adherence to the pulmonary rehabilitation plan. The observation group received CoCM-based nursing care, in addition to the standard care provided to the control group. Between days One and Three of hospitalization, the CoCM team, including healthcare providers, the patient, and family members, jointly discussed and developed a personalized postoperative pulmonary rehabilitation plan. During hospitalization, the CoCM team held regular meetings to review and refine the care plan. These sessions involved Q&A, health lectures, and case discussions to enhance communication, optimize implementation strategies, and ensure effective delivery of pulmonary rehabilitation measures. A dedicated rehabilitation record was established for each patient to document their clinical status and track progress with the rehabilitation exercises in real time. The CoCM included health education, functional training, psychological support, and peer education. Mean follow up period was six months.

### Outcome measures:

- ***Pulmonary Function Parameters:*** Forced expiratory volume in 1 second (FEV_1_), percentage of predicted FEV_1_ value (FEV_1_%), forced vital capacity (FVC), and the FEV_1_/FVC ratio were measured in both groups before and after the intervention.
- ***Arterial Blood Gas (ABG) Parameters:*** Arterial blood samples were collected pre- and post-intervention to assess partial pressure of oxygen (PaO_2_), arterial oxygen saturation (SaO_2_), and partial pressure of carbon dioxide (PaCO_2_).
- ***Functional Assessments:*** The six-minutes’ walk test (6MWT) and the Functional Assessment of Cancer Therapy-Lung (FACT-L) scale were used to evaluate patients’ activities of daily living (ADL) before and after the intervention in both groups.
- ***Quality of Life Assessment:*** The St. George’s Respiratory Questionnaire (SGRQ) was used to assess quality of life, with higher scores indicating poorer health-related quality of life.

### Statistical analysis:

All statistical analyses were performed using SPSS 22.0. According to the data of each indicator in the pre-survey, the sample size is estimated by 95% confidence interval, and the largest one is the sample size of the study. The sample size required for each group was ≥40 cases on the basis of Fisher exact probability. Continuous variables were expressed as mean ± standard deviation (*x̅*+*s*), and comparisons before and after intervention were conducted using paired t-tests. Categorical variables were expressed as frequency and percentage (*n*[%]), and intergroup comparisons were performed using the chi-square (χ^2^) test. A P-value <0.05 was considered statistically significant.

## RESULTS

According to the inclusion and exclusion criteria, a total of 80 patients were enrolled in the study, with 40 patients in each group. In the observation group, there were 22 males and 18 females, aged 40-78 years (mean: 56.35 ± 8.23 years). In the control group, there were 23 males and 17 females, aged 38-77 years (mean: 57.35 ± 8.32 years). There were no statistically significant differences between the two groups in age, sex distribution, TNM staging, or smoking status (all *P >* 0.05), indicating comparability between groups Table-I.

**Table-I:** Comparison of baseline characteristics between the two groups.

Group	n	Age (years) (x̄+s)	Sex (M/F, n)	TNM stage			Smoker (Y/N, n)
				Stage I	Stage II	Stage III	
Observation	40	56.35±8.23	22/18	9	23	8	24/16
Control	40	57.35±8.32	23/17	10	22	8	26/14
*t*/*χ*² *value*		0.540	0.051	0.075			0.213
*P-value*		0.590	0.822	0.963			0.644

Before the intervention, no significant differences in FEV_1_ FEV_1_ %, or FVC were observed between the two groups (all *P >* 0.05). At seven days, one month, and three months post-intervention, all pulmonary function parameters showed progressive improvement in both groups. However, improvements in the observation group were significantly more pronounced than in the control group at each time point (*P <* 0.05, respectively) Table-II.

**Table-II:** Comparison of pulmonary function parameters between the two groups (*x̅*+*s*), n = 40.

Parameter	Time Point	Observation	Control	t-value	P-value
FEV_1_ (L)	Pre-intervention	1.35±0.27	1.32±0.30	0.401	0.690
	At 7 days post-intervention	1.74±0.26	1.51±0.29	3.687	<0.001
	At 1 month post-intervention	2.17±0.23	1.71±0.24	8.705	<0.001
	At 3 months post-intervention	2.53±0.21	1.91±0.23	12.569	<0.001
FEV_1_%	Pre-intervention	41.18±2.65	41.69±3.38	0.747	0.457
	At 7 days post-intervention	48.09±2.62	45.61±3.29	3.736	<0.001
	At 1 month post-intervention	56.93±2.66	51.53±2.94	8.608	<0.001
	At 3 months post-intervention	63.74±3.29	56.16±4.43	8.695	<0.001
FVC (L)	Pre-intervention	2.08±0.37	1.99±0.39	1.110	0.270
	At 7 days post-intervention	2.37±0.36	2.16±0.39	2.527	0.014
	At 1 month post-intervention	2.71±0.36	2.33±0.38	4.515	<0.001
	At 3 months post-intervention	2.83±0.40	2.51±0.31	4.125	<0.001

There were no significant differences in PaO_2_, SaO_2_, or PaCO_2_, levels between the two groups before the intervention (all *P >* 0.05). At seven days, one month, and three months post-intervention, all ABG parameters showed progressive improvement in both groups. At each time point, the observation group demonstrated significantly better improvements in PaO_2_, SaO_2_, and PaCO_2_, compared with the control group (*P <* 0.05, respectively) Table-III.

**Table-III:** Comparison of ABG parameters between the two groups (*x̅*+*s*).

Parameter	Time Point	Observation	Control	t-value	P-value
PaO_2_ (mmHg)	Pre-intervention	61.79±19.15	55.57±13.03	1.697	0.094
	At 7 days post-intervention	67.23±19.77	58.87±13.42	2.213	0.030
	At 1 month post-intervention	74.99±18.05	63.54±12.63	3.286	0.002
	At 3 months post-intervention	87.98±21.0	71.78±31.97	2.678	0.009
SaO_2_ (%)	Pre-intervention	62.36±1.09	62.44±2.06	0.202	0.840
	At 7 days post-intervention	67.46±1.19	65.41±2.18	5.212	<0.001
	At 1 month post-intervention	77.38±2.92	70.66±2.45	11.135	<0.001
	At 3 months post-intervention	97.33±1.09	95.37±2.09	5.274	<0.001
PaCO_2_ (mmHg)	Pre-intervention	55.43±3.85	53.32±4.49	0.128	0.899
	At 7 days post-intervention	46.51±3.65	49.51±4.43	3.305	0.001
	At 1 month post-intervention	38.68±3.40	44.78±4.43	6.906	<0.001
	At 3 months post-intervention	33.43±2.87	36.37±6.10	2.748	0.007

Before the intervention, there were no significant differences in ADL between the two groups (*P >* 0.05). At one month and three months post-intervention, both groups showed marked improvement. Notably, the observation group exhibited significantly more pronounced enhancements in the 6MWT and the FACT-L score compared with the control group (*P <* 0.05, respectively) Table-IV.

**Table-IV:** Comparison of ADL between the two groups (*x̅*+*s*).

Group	6MWT (m)			FACT-L score		
	Pre-intervention	At 1 month post-intervention	At 3 months post-intervention	Pre-intervention	At 1 month post-intervention	At 3 months post-intervention
Observation (n = 40)	192.18±2.06	348.78±1.19	469.98±5.42	73.90±2.02	49.05±1.30	28.88±0.99
Control (n = 40)	192.13±1.32	299.50±6.40	421.05±7.48	74.63±1.66	50.60±1.34	30.93±0.92
*t-value*	0.129	47.888	33.486	1.753	5.259	9.599
*P-value*	0.897	<0.001	<0.001	0.886	<0.001	<0.001

No statistically significant difference was observed in SGRQ scores between the two groups before the intervention (*P >* 0.05). At one month and three months post-intervention, both groups demonstrated significant improvements in SGRQ scores compared with baseline. The observation group exhibited a significantly greater degree of improvement than the control group at both time points (*P <* 0.05) Table-V.

**Table-V:** Comparison of SGRQ scores between the two groups (*x̅*+*s*).

Group	n	Pre-intervention	At 1 month post-intervention	At 3 months post-intervention
Observation	40	68.93±4.37	45.78±4.25	33.80±4.28
Control	40	68.13±3.01	52.15±2.93	38.20±3.00
*t-value*		0.953	7.815	5.326
*P-value*		0.343	<0.001	<0.001

## DISCUSSION

Our study results showed significant improvements in pulmonary function and ABG parameters in both groups following intervention, with outcomes in the observation group being significantly better than in the control group (*P <* 0.05). These findings indicate that CoCM outperforms standard care in enhancing postoperative pulmonary function in patients with lung cancer. Compared with standard care, CoCM offers several distinct advantages. First, health education under this model helps patients adopt a more proactive and optimistic attitude toward their condition, improving disease awareness and treatment adherence.^6^ Second, targeted pulmonary rehabilitation guidance maximizes alveolar inflation, facilitates the clearance of airway secretions, prevents airway obstruction, and maintains airway patency.^7^,^8^ Postoperative functional exercises play a crucial role in promoting lung re-expansion and accelerating the recovery of pulmonary function.^9^ Within the CoCM framework, patients undergo a structured, iterative process of pulmonary rehabilitation, consisting of instruction, implementation, feedback, and re-instruction, aimed at reinforcing consistent and effective respiratory exercises. This approach not only strengthens patients’ adherence to daily pulmonary rehabilitation routines but also actively involves family members in supervising exercise performance. Family participation helps healthcare providers better understand and evaluate patient status, enabling the development of individualized functional training plans tailored to each patient’s needs.^10^

Lung cancer remains one of the leading causes of cancer incidence and mortality worldwide. For patients with early-stage lung cancer, surgical resection of the diseased lung tissue is the preferred treatment and, in some cases, may offer the potential for clinical cure.^11^,^12^ However, lung cancer surgery is highly invasive and often results in significant postoperative impairment of pulmonary function. Moreover, the majority of patients with lung cancer are elderly, a population frequently affected by reduced respiratory muscle strength, airway narrowing, and diminished clearance of airway secretions. This entails an increased risk of postoperative complications in elderly patients, thereby complicating both postoperative care and rehabilitation.^13^ Given these challenges, the implementation of effective nursing interventions in the postoperative period is critical for reducing complications and improving patient outcomes.

In the present study, both groups demonstrated significant improvements in the 6MWT, FACT-L, and SGRQ following the intervention (*P <* 0.05, respectively), with the observation group showing significantly more pronounced improvement than the control group (*P <* 0.05, respectively). These findings suggest that CoCM can help enhance both ADL and quality of life in patients after lung cancer surgery. All these might be explained by the following mechanisms: CoCM encourages patients to adopt a more optimistic outlook toward their disease and recovery process.^14^,^15^ Guided breathing exercises improve respiratory function, reduce the incidence of pulmonary complications, and enhance ventilation. Following intervention, patients reported reduced pain, anxiety, and fear, along with improved motivation for postoperative rehabilitation and treatment adherence.^16^

Lung cancer is a consumptive disease, and surgical intervention is a traumatic process that exacerbates physiological stress and energy depletion. Postoperatively, most patients experience varying degrees of pain, leading to reduced pulmonary and physical function, which in turn increases the risk of complications and negatively impacts quality of life.^17^,^18^ Moreover, successful recovery following lung cancer surgery requires patients to eventually reintegrate into family and social life.^19^ The ability to perform essential daily activities is a key indicator of postoperative recovery and functional independence.^20^

### Limitations:

Despite all this, the current study has several limitations. It was a single-center, retrospective analysis with a modest sample size and limited generalizability.

## CONCLUSIONS

The implementation of CoCM for postoperative patients with lung cancer can significantly promote pulmonary function recovery, improve ABG parameters, and enhance ADL and quality of life. Given its benefits, this care model is recommended for broader clinical application.

### Recommendations:

Future research should incorporate large-scale, multi-center, prospective studies to validate and extend these findings.

### Authors’ Contributions:

**PL:** Conception and the design of the study, editing of manuscript, is responsible for integrity of research.

**CL** and **WL:** Acquisition, analysis and interpretation of the data.

**XQ, CS** and **HC:** Revised it critically. Literature review

All authors have read and approved the final manuscript.
